# Learning about Enzyme Stability against Organic Cosolvents from Structural Insights by Ion Mobility Mass Spectrometry

**DOI:** 10.1002/cbic.201900648

**Published:** 2020-03-05

**Authors:** Jens Sproß, Yasunobu Yamashita, Harald Gröger

**Affiliations:** ^1^ Chair of Industrial Organic Chemistry and Biotechnology Faculty of Chemistry Bielefeld University Universitätsstrasse25 33615 Bielefeld Germany

**Keywords:** ene reductase, enzyme catalysis, ion mobility spectrometry, mass spectrometry, protein structures

## Abstract

Ion mobility spectrometry (IMS) coupled with mass spectrometry (MS) enables the investigation of protein folding in solution. Herein, a proof‐of‐concept for obtaining structural information about the folding of a protein in dependency of the amount of an organic cosolvent in the aqueous medium by means of this IMS‐MS method is presented. By analyzing the protein with native nano‐electrospray ionization IMS‐MS, the impact of acetonitrile as a representative organic cosolvent and/or pH values on the folding of an enzyme was successfully evaluated in a fast and straightforward fashion, as exemplified for an ene reductase from *Gluconobacter oxydans*. The IMS‐MS results are in agreement with findings from the nicotinamide adenine dinucleotide phosphate (NADPH)‐based spectrophotometric enzyme activity tests under analogous conditions, and thus, also rationalizing these “wet” analytical data. For this ene reductase, a higher tolerance against CH_3_CN in the presence of a buffer was observed by both analytical methods. The results suggest that this IMS‐MS methodology could be a useful complementary tool to existing methods in process optimization and fine‐tuning of solvent conditions for biotransformations.

## Graphical abstract

**Aqueous origami**: Structural information about the folding of a protein, depending on the amount of acetonitrile as organic cosolvent in the aqueous medium, is obtained through ion mobility mass spectrometry for an ene reductase from *G. oxydans*. These results are in agreement with findings from spectrophotometric enzyme activity tests under analogous conditions.

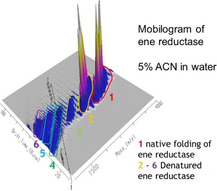

In recent decades, an increasing tendency to apply biocatalysis in organic synthesis was observed.^1^, ^2^ Due to advantages, such as excellent selectivity and catalytic activity, enzyme catalysis also gained tremendous interest from the chemical industry, in particular, for the production of fine chemicals and pharmaceuticals.^1^, ^2^ In developing organic synthetic processes with enzymes, often the use of water‐miscible organic solvents are considered as an option because the solubility of substrates (which are hydrophobic, in many cases, in organic chemistry) in water as the solvent of choice for enzymes can be increased.^1^ It is known that proteins have the potential to keep their function in buffered aqueous solutions containing an amount of such an organic solvent. On the other hand, activity and, in particular, stability of enzymes can severely be affected by the presence of organic cosolvents. Thus, during biocatalytic process development, typically the impact of an organic solvent on enzyme activity and, in particular, enzyme stability is investigated. To screen such effects, a ready‐to‐use assay with a simple read‐out is desired. As for the field of redox enzymes, which are the second mostly applied enzyme class in organic synthesis after hydrolases, often such an assay depends on spectrophotometry. Measuring the decrease or increase of NAD(P)H is a widely used tool for the determination of activities of, for example, dehydrogenases, which are dependent on such cofactors. As an efficient method for obtaining information on activity, at the same time, it would be desirable to rationalize these findings on activity by gaining an insight into structural changes of the proteins under the same experimental conditions. Encouraged by the success of ion mobility mass spectrometry (IMS) coupled with mass spectrometry (MS) for studying protein structures in the absence of bulk water,^3^, ^4^ we became interested in studying IMS‐MS methodology^5^, ^6^ for obtaining information about the folding of this protein in dependency of the amount of an organic cosolvent in the aqueous medium. The results on the folding properties in the gas phase are expected to give an insight into the solvent effects of the enzyme in the condensed phase. Herein, we report our results on such a comparison of the structural information about the enzyme obtained from IMS (after treatment of the enzyme with an organic cosolvent) with those of the “wet” analytical data obtained from the standard spectrophotometric measurement of the enzyme activity under the same experimental conditions.

As a “model enzyme” for the enzyme class of redox enzymes, we chose the ene reductase from *Gluconobacter oxydans*, which turned out to represent a versatile biocatalyst that is useful for the reduction of different types of activated C=C double bonds.^7^, ^8^, ^9^, ^10^ Furthermore, a broad screening of different reaction conditions has been performed with this enzyme previously, from which a range of data for the impact of water‐soluble organic cosolvents are available.^7^, ^8^, ^9^, ^10^ As a representative solvent for the water‐soluble organic cosolvent in the aqueous buffer solution, we selected acetonitrile for our study because, in earlier studies, the ene reductase from *G. oxydans* showed some, but, at the same time, limited stability if using this solvent, CH_3_CN, under certain conditions.^10^ Thus, CH_3_CN appeared to us to be a preferred solvent for this study because remaining active enzyme as well as some deactivation effects can be expected, which then should also be observed by MS analysis. In addition, CH_3_CN is of interest as a cosolvent for other enzymes. For example, cleavage of proteins by using immobilized trypsin in the presence of up to 20 % CH_3_CN yielded slightly higher sequence coverages compared with that of pure water solutions,^11^ and monomeric avidin was able to bind biotinylated peptides in the presence of up to 20 % CH_3_CN.^12^

In our study, using IMS, we were also interested in gaining an insight into whether the decrease in activity was related to changes in enzyme folding caused by CH_3_CN or due to the pH of the aqueous solution. A qualitative evaluation of the IMS‐MS spectra is sufficient because the ionization efficiencies of different protein folding states are likely to be similar. Toward this end, the ene reductase from *G. oxydans* in purified form was treated with various solvent systems containing a buffered or unbuffered aqueous medium with different amounts of CH_3_CN. Subsequently, such mixtures were analyzed by using a nano‐ESI‐Q‐IMS‐*oa*‐TOF mass spectrometer (nano‐electrospray quadrupole‐ion mobility spectrometry orthogonal acceleration time‐of‐flight; Synapt G2Si, Waters, Manchester) under native conditions.

In ESI‐MS experiments, the range of compatible buffers is limited because most commonly used buffers, such as phosphate buffer, cause clogging of the mass spectrometer. The protein concentration in these experiments was 10 μm of purified ene reductase, and measurements were performed either in unbuffered water or in 0.1 m ammonium acetate buffer at a pH value of 6.2. The percentage of CH_3_CN ranged from 0 to 35 vol %. The IMS separation was performed by using nitrogen as the drift gas, at a wave velocity of 500 m s^−1^ and a wave height of 25 V.

When we analyzed the ene reductase from an unbuffered aqueous solution with 5 % CH_3_CN, the mobilogram revealed the presence of six different folding states, including protein in a native folding and completely denatured protein (Figure 1 A). If the experiment was conducted by using an unbuffered solution of ene reductase in water, the degree of unfolding was virtually identical (Figure S1 in the Supporting Information). The noncovalently bound FMN cofactor remained only partially in complex with the ene reductase in the native‐like folding state (Figure 1 B). Therefore, the cofactor does not have a stabilizing effect on the ene reductase and is easily lost in the presence of small amounts of CH_3_CN in unbuffered solution. Unfolding of the ene reductase and loss of the FMN cofactor suggest a drop in enzyme activity.

**Figure 1:**
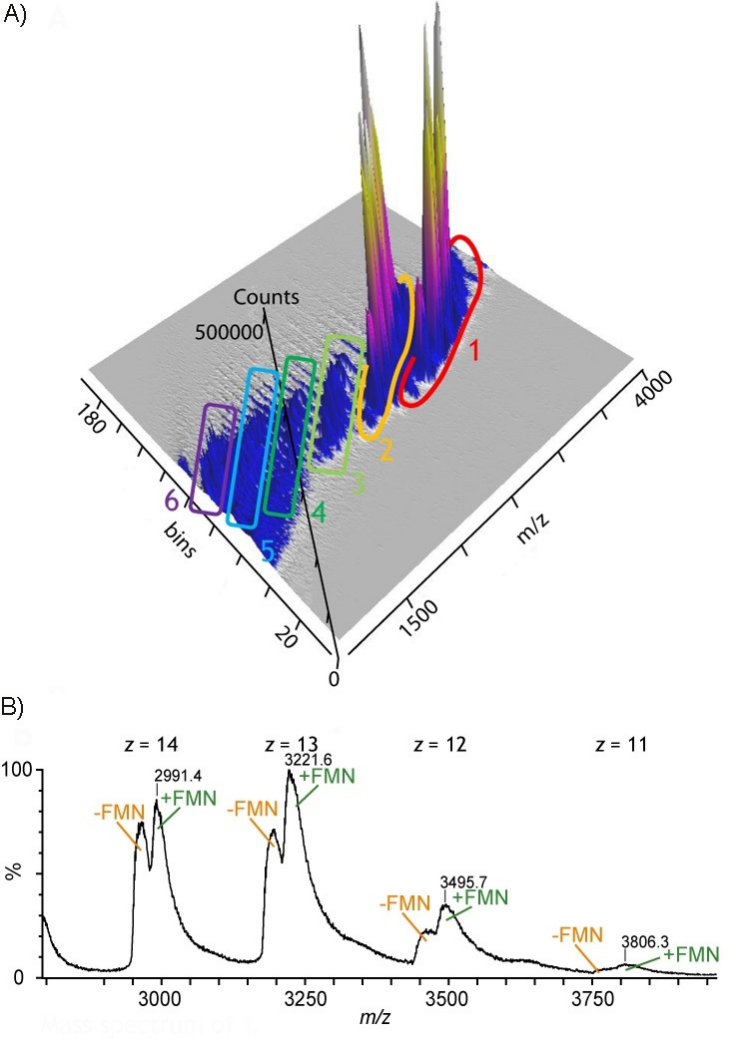
A) Mobilogram of ene reductase, obtained from an aqueous solution containing 5 % CH_3_CN, revealing the presence of up to six different folding states. The pH value of this solution was determined to be 8.6. B) Mass spectrum of folding state 1, corresponding to the native folding of ene reductase. Ene reductase is present in complex with its flavin mononucleotide (FMN) cofactor and without the cofactor.

Interestingly, mobilograms obtained by measurements performed from solutions of ene reductase in 0.1 m ammonium acetate (pH 6.2) showed that the amount of CH_3_CN could be raised up to 30 %, with only small signs of unfolding of the protein (Figures 2 A, S2, and S3). The FMN cofactor was still bound to the ene reductase, despite the high amounts of CH_3_CN present in the mixture (Figure 2 B). Increasing the CH_3_CN content above 30 % initiated unfolding of the protein (Figure S4). The same results were obtained with a pH value of 7.2, buffered by ammonium acetate. These results suggest that, in buffered solution, ene reductase retains its catalytic activity, even in the presence of higher amounts of CH_3_CN, in contrast to the unbuffered solution.

**Figure 2:**
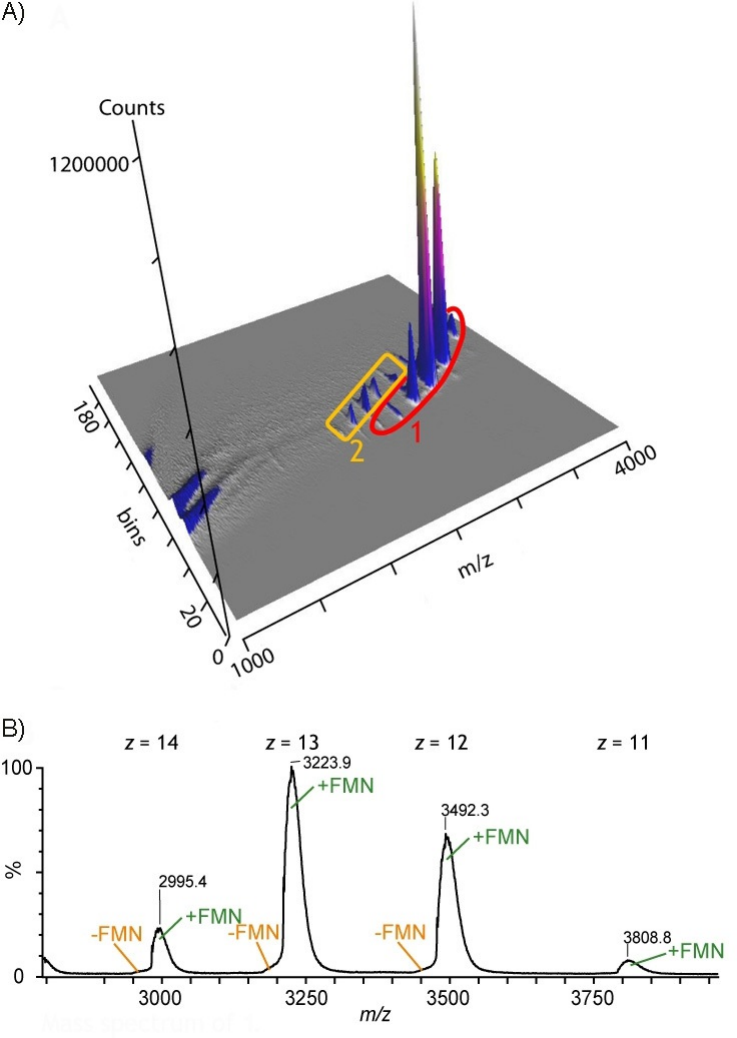
A) Mobilogram of ene reductase, obtained from a buffered solution (0.1 m NH_4_Ac, pH 6.2) containing 25 % CH_3_CN, showing mainly the native folding state 1 and a small amount of partially unfolded ene reductase (2). B) Mass spectrum of folding state 1, corresponding to the native folding of ene reductase, almost exclusively in complex with its cofactor FMN.

To validate such results from the IMS‐MS study, as a next step, we performed spectrophotometric determinations of the activity of ene reductase by recording the change in absorbance upon oxidation of the nicotinamide adenine dinucleotide phosphate (NADPH) cofactor after treatment of the enzyme with CH_3_CN under analogous conditions to those in the IMS‐MS study. Through these experiments, we were interested in verifying the findings made in the MS study. In detail, we were interested in whether the results from the spectrophotometric determination of the enzyme activity confirmed the hypotheses made from the protein folding results of the IMS‐MS experiments—in the presence of CH_3_CN, the enzyme activity is retained in buffered solution and is lost in unbuffered solution. The enzymatic activity of the ene reductase from *G. oxydans* was determined in unbuffered or buffered solutions containing various amounts of CH_3_CN. For the determination of the enzyme activity, citral was used as a standard substrate and the oxidation of NADPH to NADP^+^ was measured by the decrease in the absorbance at *λ*=340 nm and room temperature. The principle of the setup of this spectrophotometric enzyme activity assay is shown in Scheme 1.

**Scheme 1:**
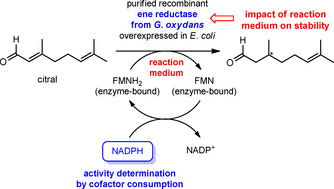
Principle of the spectrophotometric determination of the enzyme activity of the ene reductase from *G. oxydans* in the absence or presence of an organic cosolvent.

The reaction conditions, such as protein and buffer concentration and amount of CH_3_CN as a cosolvent (0 to 40 vol %), were adjusted to those of the IMS‐MS experiments. The results of this spectrophotometric study are shown in Figure 3. In the unbuffered system, a steady decrease in enzyme activity was observed with increasing CH_3_CN content. Even the lowest amount of CH_3_CN (2.5 %) had a negative effect on the activity of the ene reductase and higher CH_3_CN contents resulted in a stronger drop of activity.

**Figure 3:**
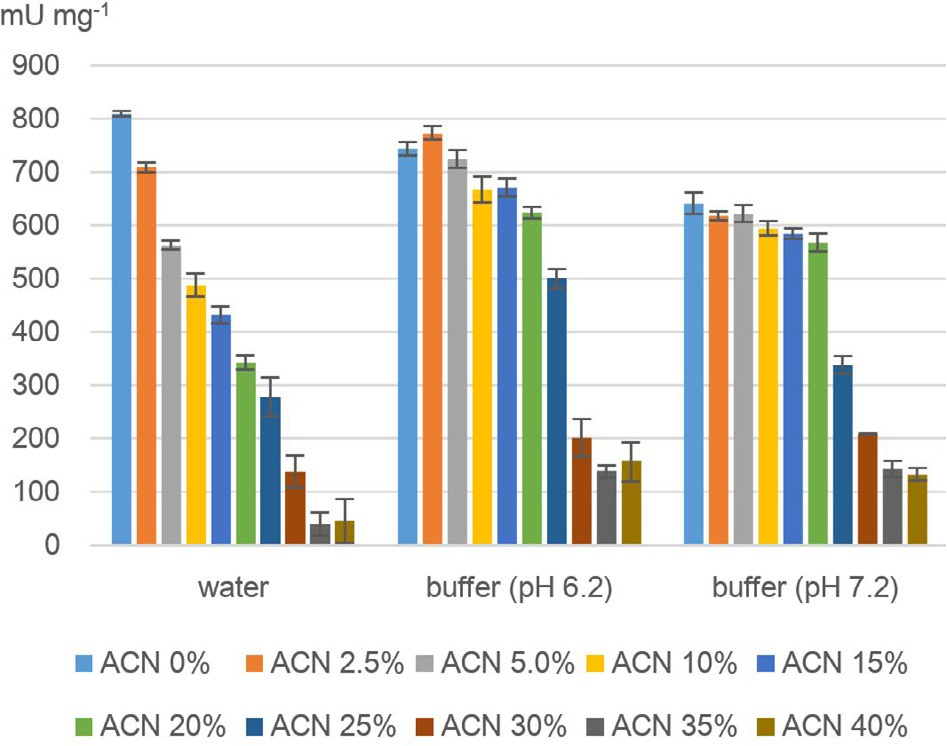
Spectrophotometric determination of the enzyme activity using unbuffered or buffered aqueous solutions in the presence of different amounts of CH_3_CN (0–40 %).

On the other hand, we observed a higher tolerance against CH_3_CN in the presence of 0.1 m ammonium acetate buffer (both pH 6.2 and 7.2), showing a relatively high remaining activity with up to about 25 % CH_3_CN cosolvent in case of buffer at pH 6.2 and up to about 20 % in case of the buffer at pH 7.2 (Figure 3).

It is noteworthy that these findings were in good agreement with the IMS‐MS experiments and the observed folding states of the enzyme at various amounts of the cosolvent CH_3_CN. For example, the high remaining activity of the enzyme, even in the presence of 25 % CH_3_CN, found in the spectrophotometric study by using buffer at pH 6.2 (Figure 3) is in accordance with the IMS‐MS result, showing the mainly native folding state under this condition (Figure 2 A).

In conclusion, we could demonstrate a proof‐of‐concept for obtaining structural information about the folding of a protein in dependency of the amount of an organic cosolvent in the aqueous medium by means of IMS‐MS. By analyzing the protein with native nano‐ESI‐IMS‐MS, the impact of CH_3_CN as an organic cosolvent and/or pH values on the folding of an enzyme was successfully evaluated in a fast and straightforward fashion exemplified for an ene reductase from *G. oxydans*. These IMS‐MS results are in agreement with the findings from the spectrophotometric enzyme activity tests under analogous conditions, and thus, also rationalize these wet analytical data. For example, for the ene reductase from *G. oxydans*, a higher tolerance against CH_3_CN in the presence of a buffer was observed by both analytical methods. According to IMS‐MS, unfolding of ene reductase increased in unbuffered systems with increasing percentage of CH_3_CN. In addition, this IMS‐MS procedure represents a fast and reliable approach for the identification of organic cosolvent effects on the enzyme structure and thereby the enzymatic activity, which is a valuable information for improving biotransformations. Although the range of compatible buffers is limited in ESI‐MS experiments because of clogging of the mass spectrometer, this IMS‐MS methodology can be considered as a complementary tool to other existing methods (e.g., spectrophotometric assays) for a screening towards the identification of suitable reaction conditions for biotransformations with cofactor‐containing enzymes. Further studies with other enzymes by using this IMS‐MS methodology for biocatalysis process development are currently in progress in our group, and we hope that this method will find broad utilization in the biocatalysis community for process development and, for example, identification of suitable water‐organic reaction media systems for biotransformations. In addition, in future work, we also will focus on gaining an insight into the structures of the various folding states of the enzyme, resulting from the treatment with an organic solvent.

## Experimental Section

**Enzyme preparation**: Preparation of purified recombinant N‐terminally hexahistidine‐tagged ene reductase from *G. oxydans* was performed as described previously and involved the transformation of *Escherichia coli* Bl21(DE3) cells with the expression plasmid pGOX, subsequent fermentation, and purification after cell disruption through immobilized‐metal affinity chromatography (IMAC; Ni‐NTA).^7^, ^8^, ^9^, ^10^

**Spectrophotometric determination of the enzyme activity**: In this enzyme activity assay, the oxidation of NADPH to NADP^+^ was measured by monitoring the decrease in absorbance at *λ*=340 nm at room temperature. The enzyme activity is defined as mU mg^−1^ by using Equation (1) and enzyme concentration:

(1) $$A=\frac{\Delta EV}{\epsilon dv}$$

in which *A* is the enzyme activity (U mL^−1^), Δ*E* is the absorption change in min^−1^, *V* is the total sample volume, *ϵ* is the excitation coefficient (6.3 mL mmol^−1^ cm^−1^) of NADPH at *λ*=340 nm, *d* is the layer thickness in microtiter plates, and *v* is the volume of enzyme solution. The reaction was performed in a microtiter by using a TecanReader (TECAN, Männedorf, Switzerland) system at room temperature with a volume of 200 μL. Purified ene reductase (10 μm) was added to a solution of citral (2.5 mm) in water or ammonium acetate buffer (0.1 m, pH 6.2 or 7.2) in the presence of 0 to 40 % CH_3_CN, and incubated at room temperature for 20 min, then NADPH (0.4 mm) was added to measure the enzyme activity.

**IMS‐MS analysis**: Nano‐ESI‐IMS‐*oa*‐TOF mass spectra (*m*/*z* 50–5000) were recorded in resolution mode and positive‐ion mode with a Synapt G2Si spectrometer (Waters Corp., Manchester, UK). Ene reductase at a concentration of 10 μm was dissolved in water in the presence of 0 to 5 % CH_3_CN or in 0.1 m NH_4_Ac, pH 6.2, containing 0 to 35 % CH_3_CN. Sample solutions were introduced by static nano‐ESI by using in‐house‐pulled glass emitters. A voltage of 1 kV was applied to the nano‐ESI emitter. Nitrogen served both as the nebulizer gas and the dry gas for nano‐ESI and was generated by a nitrogen generator NGM 11. The source settings were as follows: sampling cone 25 V, source offset 55 V, source temperature 40 °C, cone gas flow 20 L h^−1^, nanoflow gas pressure 0.4 bar, and nebulizer gas pressure 6 bar. To enable soft transport of ions through the instrument, the trap collision cell voltage was set to 1.5 V and a flow of 2 mL min^−1^ of argon 5.0; the transfer collision cell was operated at 0.5 V. The instrument was equipped with a traveling‐wave IMS cell. Helium 5.0 was used as buffer gas in the IMS entry cell; nitrogen generated by nitrogen generator NGM 11 was used for IMS separations. The gas flow in the helium cell prior to IMS was set at 180 mL min^−1^ and the IMS separation occurred at a nitrogen gas flow of 70 mL min^−1^, resulting in a pressure of about 4 mbar in the helium cell and 2.6 mbar in the IMS cell. The IMS voltage settings were as follows: IMS direct current (DC) entrance 20 V, helium DC 50 V, helium exit −20 V, and IMS bias 25 V. The IMS separation was performed at a wave velocity of 500 m s^−1^ and a wave height of 25 V. Mass spectra were recorded for 120 s with a scan time of 1 s. Data were collected by using MassLynx 4.1 software. External calibration of the instrument was performed by using ESI‐L Tuning Mix (Agilent Technologies, Santa Clara, CA, USA) as the calibration standard. Data analysis and processing were performed by using MassLynx 4.1 and Driftscope v2.9 software (Waters Corp., Manchester, UK).

## Conflict of interest

*The authors declare no conflict of interest*.

## Supporting information

As a service to our authors and readers, this journal provides supporting information supplied by the authors. Such materials are peer reviewed and may be re‐organized for online delivery, but are not copy‐edited or typeset. Technical support issues arising from supporting information (other than missing files) should be addressed to the authors.

Supplementary
