# Supplementary material for: Learning about Enzyme Stability against Organic Cosolvents from Structural Insights by Ion Mobility Mass Spectrometry
Source: Chembiochem. 2020 Mar 5;21(14):1968–71. doi: 10.1002/cbic.201900648 (PMC7496688; doi:10.1002/cbic.201900648)
Supplement: Supplementary file 1 — Supplementary [file CBIC-21-1968-s001.pdf]

CHEMBIOCHEM

Supporting Information

Learning about Enzyme Stability against Organic Cosolvents from Structural Insights by Ion Mobility Mass Spectrometry

Jens Sproß,* Yasunobu Yamashita, and Harald Gröger*[a]

cbic_201900648_sm_miscellaneous_information.pdf

Supporting Information:

Table of Content

#	Content	page
1	Materials	3
FigS1	IMS-MS results of ene reductase in water, containing 0% ACN	4
FigS2	IMS-MS results of ene reductase in 0.1 M NH_4Ac, pH 6.2, containing 0% ACN	5
FigS3	IMS-MS results of ene reductase in 0.1 M NH_4Ac, pH 6.2, containing 5% ACN	6
FigS4	IMS-MS results of ene reductase in 0.1 M NH_4Ac, pH 6.2, containing 35% ACN	7

Materials

Ammonium acetate was purchase from Sigma Aldrich (St. Louis, MO, USA) and used without further purification.

Water was purified with a Millipore System.

Acetonitrile was obtained in UPLC-MS grade from BioSolve (Valkenswaard, Netherlands).

Citral was purchase from Alfa Aesar GmbH & Co KG (Karlsruhe, Germany) and used without further purification.

NADPH was purchase from Carl Roth GmbH & Co KG (Karlsruhe, Germany) and used without further purification.

Figure S1

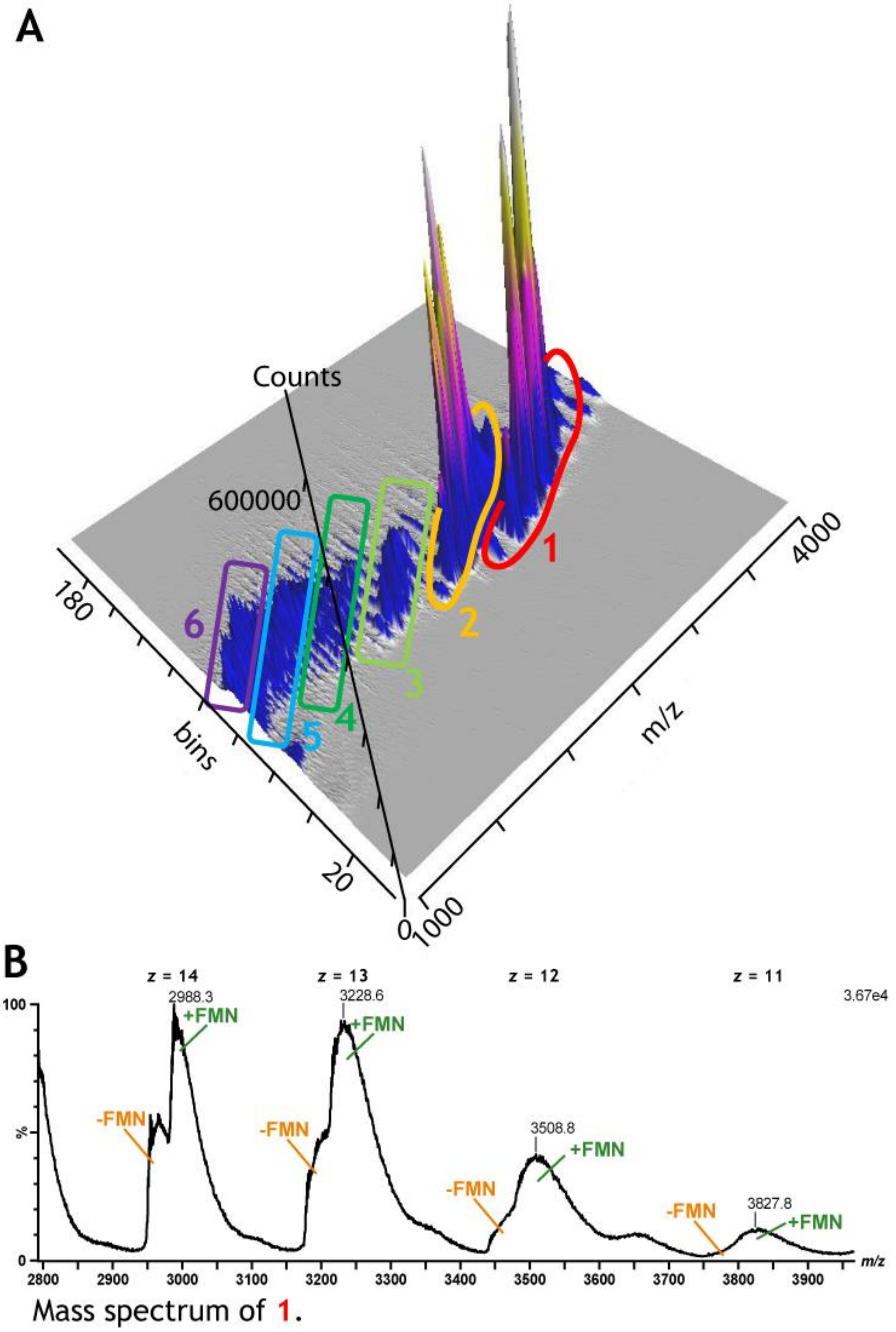

Figure S1: A Mobilogram of ene reductase, obtained from an aequeous solution containing 0% ACN, revealing the presence of up to 6 different folding states. The pH value of this solution was determined to be 8.6. **B** Mass spectrum of folding state 1, corresponding to the native folding of ene reductase. Ene reductase is present in complex with its cofactor FMN and without the cofactor.

Figure S2

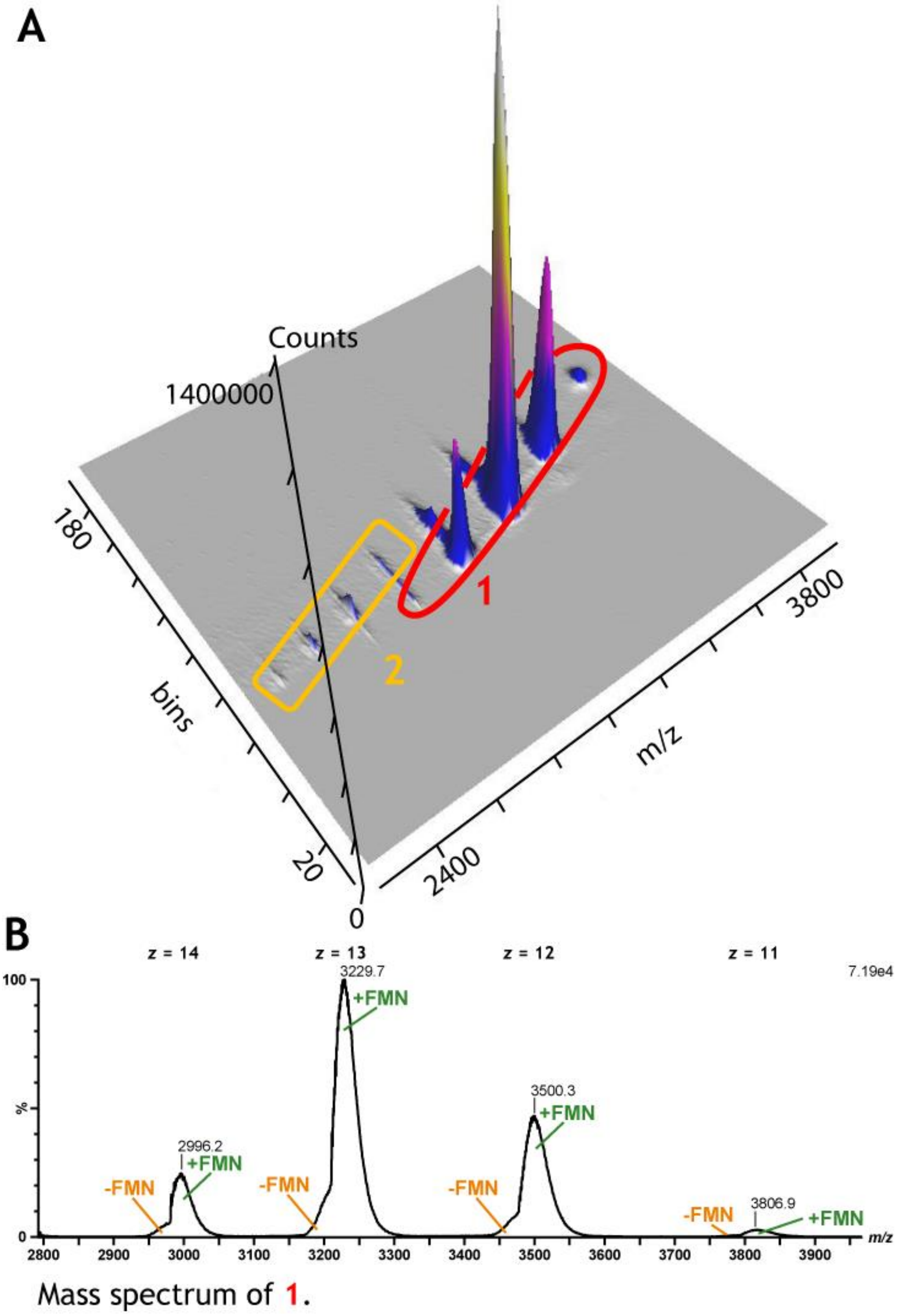

Figure S2: A Mobilogram of ene reductase, obtained from a buffered solution (0.1 M NH_4Ac, pH 6.2) containing 0% ACN, showing mainly the native folding state 1. Only low amounts of the protein are partially unfolded (folding state 2). **B** Mass spectrum of folding state 1, corresponding to the native folding of ene reductase. Ene reductase is mainly present in complex with its cofactor FMN.

Figure S3

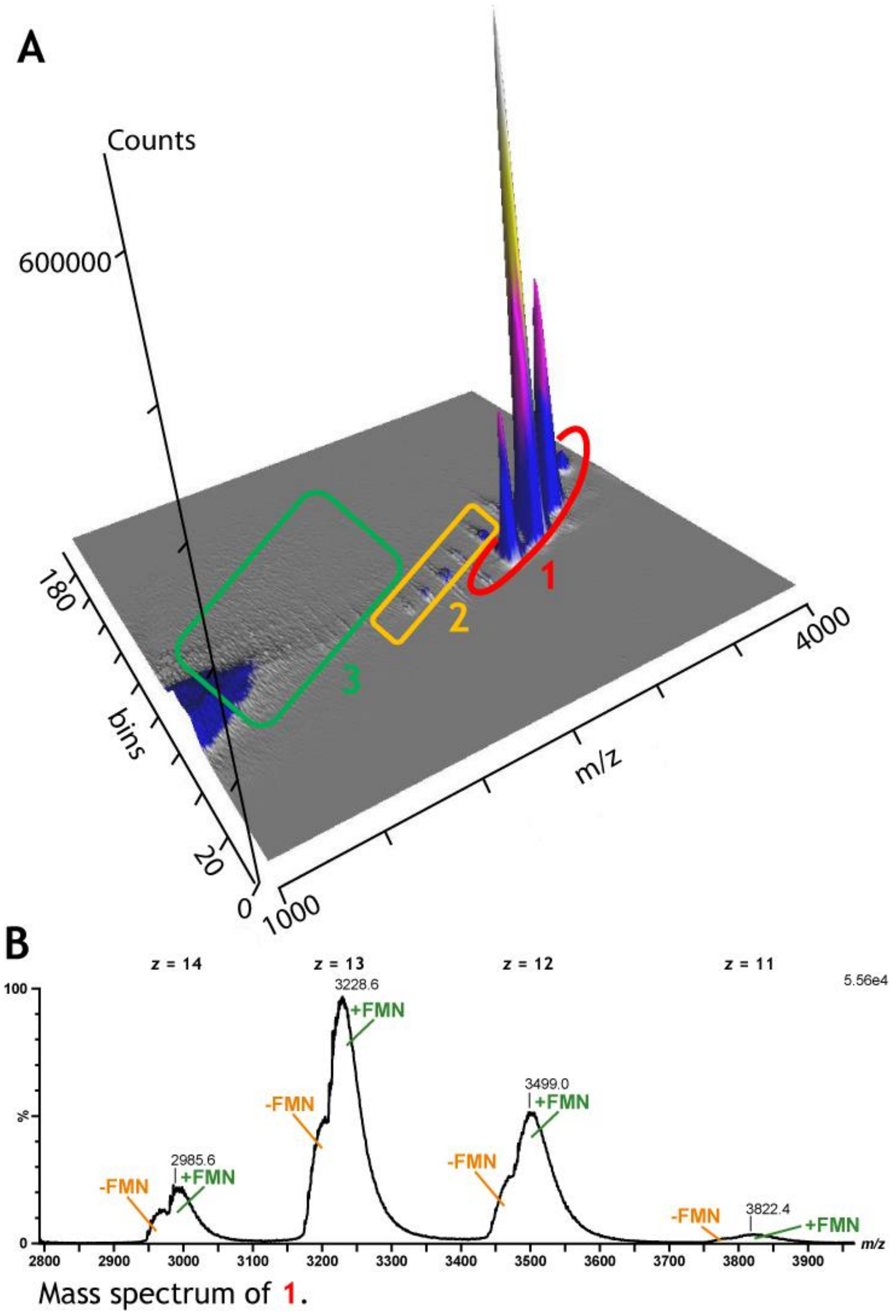

Figure S3: A Mobilogram of ene reductase, obtained from a buffered solution (0.1 M NH_4Ac, pH 6.2) containing 5% ACN, showing mainly the native folding state 1. Only low amounts of the protein are partially unfolded (folding state 2) or show a higher degree of unfolding (3). **B** Mass spectrum of folding state 1, corresponding to the native folding of ene reductase. Ene reductase is mainly present in complex with its cofactor FMN but also ene reductase without FMN can be detected.

Figure S4

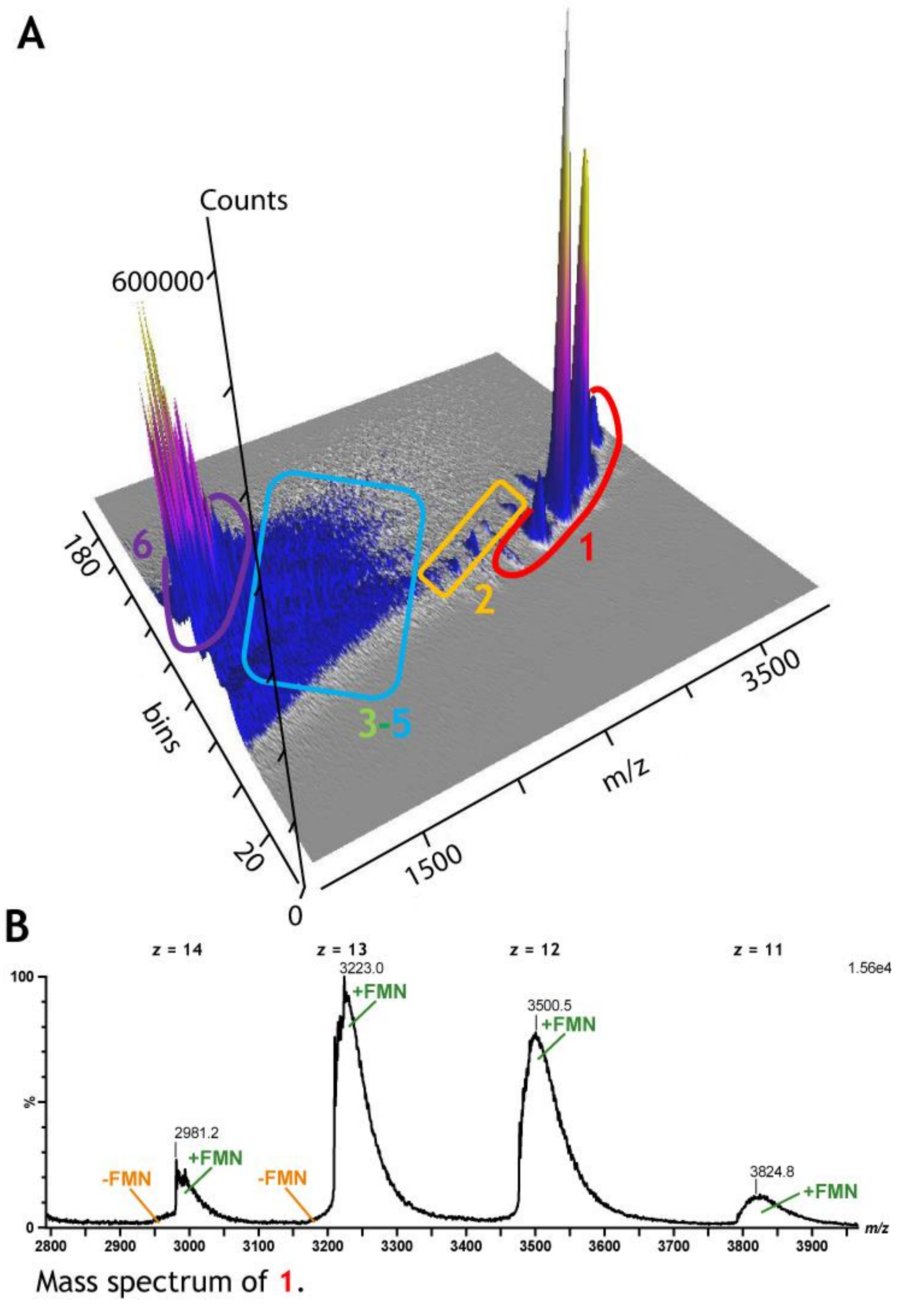

Figure S4: A Mobilogram of ene reductase, obtained from a buffered solution (0.1 M NH_4Ac, pH 6.2) containing 35% ACN, showing the native folding state 1, partially unfolded protein (folding state 2) and signals corresponding to a higher degree of unfolding (folding state 3-5). Folding state 6 corresponds to completely unfolded protein. **B** Mass spectrum of folding state 1, corresponding to the native folding of ene reductase. Ene reductase is almost exclusively present in complex with its cofactor FMN.
